# Systemic inflammation is associated with depressive symptoms differentially by sex and race: a longitudinal study of urban adults

**DOI:** 10.1038/s41380-019-0408-2

**Published:** 2019-04-24

**Authors:** May A. Beydoun, Hardeep K. Obhi, Jordan Weiss, Jose A. Canas, Hind A. Beydoun, Michele K. Evans, Alan B. Zonderman

**Affiliations:** 1 Laboratory of Epidemiology and Population Sciences, National Institute on Aging, NIA/NIH/IRP, Baltimore, MD USA; 2 Population Studies Center and the Leonard Davis Institute of Health Economics, University of Pennsylvania, Philadelphia, PA USA; 3 Johns Hopkins All Children’s Hospital, St. Petersburg, FL USA; 4 Department of Research Programs, Fort Belvoir Community Hospital, Fort Belvoir, VA USA

**Keywords:** Predictive markers, Depression

## Abstract

Systemic inflammation may influence trajectories of depressive symptoms over time, perhaps differentially by sex and race. Inflammatory markers and the Center for Epidemiologic Studies-Depression scale [total score: CES-D_total_ and four distinctive domains: somatic complaints, depressed affect, positive affect and interpersonal problems] were examined among African-American (AA) and White urban adults participating in the Healthy Aging in Neighborhoods of Diversity across the Life Span (HANDLS) study [2004–2013, Age_base_:30–64 y, mean ± SD follow-up time: 4.64 ± 0.93 y, *N* = 150 (with cytokine data) to *N* = 1,767 (with other inflammatory markers)]. Findings suggest that serum concentrations of high-sensitivity C-reactive protein (hsCRP), z-inflammation composite score [ICS, combining elevated hsCRP and ESR with low serum albumin and iron], and serum interleukin (IL) 1β were positively associated with ΔCES-D_total_ (Δ: annual rate of increase) among Whites only. IL-12 was directly related to ΔCES-D_total_ among men and AA. The race-specific associations of hsCRP, ICS, IL-1β and the sex-specific association of IL-12 with ΔCES-D_total_ were replicated for the “depressed affect” domain. Similarly, among men, lower serum albumin and higher ICS were linked with higher baseline “somatic complaints”. IL-10 among AA and IL-12 among men were inversely related to Δ“positive affect”, while “interpersonal problems” were cross-sectionally associated with IL-6 among AA and IL-10 among Whites. Finally, baseline ICS was positively associated with incident “elevated depressive symptoms” (EDS: CES-D_total_ ≥ 16) among AA (HR = 1.28, 95% CI: 1.04–1.56, *P* = 0.017). Overall, systemic inflammation was directly linked to increased depressive symptoms over time and at baseline, differentially across sex and race groups. More longitudinal research is needed to replicate our findings.

## Introduction

Depression is characterized by altered mood and poor cognitive function [1, 2] and is associated with a proinflammatory process which can lead to increased morbidity from cardiovascular disease [3, 4]. A number of psycho-neuroimmunological dysfunctional processes have been proposed to help elucidate the origins of depression [2]. Indeed, depressed adults are observed to have activated peripheral immune systems with exaggerated proinflammatory cytokine production and abnormalities in neurotransmitter metabolism, neuroendocrine functions and regional brain activity. These aberrant pathways could increase the number of depressive symptoms [2]. Acute cytokine administration to humans and animals triggers sickness behavior, which shares common features with depression [2]. In addition, chronic stress is associated with a proinflammatory phenotype among adults leading to recurrent depressive episodes [2].

The Center for Epidemiological Studies-Depression scale can be used to extract elevated depressive symptoms (EDS) and can quantify total and domain-specific (e.g., depressed affect *vs*. somatic complaints) depressive symptom scores in populations. Systemic inflammation can be quantified by increased proinflammatory cytokines (e.g., interleukins: IL-1β, IL-6, IL-12 and IL-18) vs. decreased anti-inflammatory cytokines (e.g. IL-10) as well as the high-sensitivity C-reactive protein (hsCRP) levels, whose expression is triggered by IL-6 [1, 5, 6]. Inflammation is also accompanied by an increased level of fibrinogen (proxied by the Erythrocyte Sedimentation Rate, ESR) [7], as well as reductions in serum transferrin saturation (and/or serum iron) and in serum albumin [8]. Importantly, depression was linked to systemic inflammation in recent large epidemiological studies [3, 9–20]. Nevertheless, longitudinal relationships between systemic inflammatory markers and depressive symptoms in urban populations are under-studied, particularly when those relationships are examined across sex, race- and domains of depressive symptoms.

Using the longitudinal data on an ethnically diverse urban sample, our study has two objectives. First, we will assess the cross-sectional (i.e., baseline vs. baseline) and longitudinal (i.e., baseline vs. annual rate of change (Δ); baseline vs. incident EDS) relationships of systemic inflammation markers with depressive symptoms. We hypothesize that depressive symptoms are worsened by systemic inflammation. Second, we examine the cross-sectional and longitudinal relationships of systemic inflammation with specific domains of depressive symptoms. All these associations will be tested separately by sex and race.

## Materials And Methods

### Database

The Healthy Aging in Neighborhoods of Diversity across the Life Span (HANDLS) study is an ongoing prospective cohort study initiated in 2004. HANDLS examines health disparities associated with race and socioeconomic status. Using an area probability design, the study recruited a representative sample of African-Americans (AA) and Whites with baseline ages 30–64 years residing in Baltimore, Maryland [21]. The baseline visit (visit 1) had two phases. Phase I conducted participant screening and recruitment and administered a general household interview. Phase II consisted of in-depth examinations in mobile Medical Research Vehicles (MRVs). Follow-up examinations were performed in the MRVs. Following access to a protocol booklet in layman’s terms coupled with a video describing procedures and re-contacts, written informed consent was obtained. The National Institute on Environmental Health Sciences Institutional Review Board of the National Institutes of Health approved the study protocol.

Our current study extracted longitudinal data from baseline (visit 1, performed in 2004–2009) designated as visit 1 and first follow-up examination (visit 2, performed in 2009–2013, mean ± SD follow-up interval = 4.65 y ± 0.93 y) designated as visit 2.

### Study Sample

The initial HANDLS sample consisted of 3,720 participants (Sample 1). Dietary data were available for *N* = 2,177 baseline participants (2 24 h dietary recalls) while depressive symptoms data were available for *N* = 2,736 and *N* = 2,239 at visits 1 and 2, respectively. Baseline systemic inflammatory marker data were complete among *N* = 2,580 [serum high sensitivity C-reactive protein (hsCRP), Erythrocyte Sedimentation Rate (ESR), albumin and iron] and among *N* = 244–259 for cytokines (interleukins, IL-1β, IL-6, IL-10, IL-12 and IL-18). For participants with complete dietary data, depressive symptoms were complete on 1 or 2 visits for *N* = 1,991 subjects. Inclusion and exclusion criteria were determined based on completeness of outcomes, exposures and covariates, with no other sample restrictions. Excluding missing data on baseline exposures and covariates along with missing data on depressive symptoms at both visits yielded two final analytic samples [Sample 2A: *N*_*2A*_ = 1,767; repeated observations *N*′_*2A*_ = 3,054 for data complete on serum hsCRP, ESR, albumin and iron; Sample 2B: *N*_*2B*_ = 150; repeated observations *N*′_*2B*_ = 260 for data complete on all cytokines]. Participants in Sample 2A differed significantly from the remaining Sample 1 of HANDLS by age ( + 0.72 y older, *P* = 0.019), with a reduced proportion of AA (56.7 vs. 61.3%, *P* = 0.005). Compared to Sample 1, Sample 2B had a higher proportion of AA (75.3 vs. 58.4%, *P* < 0.001), but lower proportions of men (35 vs. 46%, *P* = 0.008) and individuals above poverty (48 vs. 59%, *P* = 0.006). A two-stage Heckman selection model was used to adjust for potential selection bias associated with these sociodemographic factor differentials.

### Depressive symptoms

Measures of depressive symptoms at each visit were available using the 20-item Center of Epidemiological Studies-Depression (CES-D), a self-reported symptom rating scale assessing affective and depressed mood [22], which has good psychometric properties in various studies of older adults [23]. A total CES-D (CES-D_total_) score ≥ 16 reflects elevated depressive symptoms (EDS) [24] (CES-D_total_) consists of meaningful domains exhibiting an invariant factor structure between the National Health and Nutrition Examination Survey I and pilot HANDLS data [25]. Thus, our hypotheses were tested using the total score and domain-specific CES-D scores: (1) Somatic complaints; (2) Depressive affect; (3) Positive affect and (4) Interpersonal problems [25].

### Measures of systemic inflammation and composite score

Blood obtained during the MRV baseline visit was sent directly to the lab (Quest Diagnostics, Chantilly, VA) and processed on the same day or overnight. The sample was not frozen. The variance in the timing was relatively small since a courier retrieved the samples from our field site and had them delivered to the Quest Diagnostics laboratory facility directly, using the same schedule each day. As stated earlier, several blood biomarkers tend to increase during an inflammation while others tend to decrease [8]. While serum hsCRP and serum fibrinogen tend to increase, serum albumin and transferrin saturation tend to decrease with inflammation [8]. Proxies for serum fibrinogen and transferrin saturation are ESR and serum iron, respectively. Using NHANES III data, the high correlation between serum iron and serum transferrin saturation was verified (*r* = 0.92, *P* < 0.001) in a comparable sample (age range: 30–65 y). Similarly, previous studies show that ESR is highly correlated with serum fibrinogen [7]. Examining them individually, each marker was used as a continuous untransformed variable in the main analysis. By running principal components analysis, data on the 4 individual measures of inflammation (hsCRP, ESR, albumin and iron) were reduced into a single standardized measure explaining > 40% of the total variance, which we labelled “z-inflammation composite score” (ICS). Alternative PCA analyses, which included white blood cell counts, serum ferritin and transferrin saturation among others confirmed that this ICS was the most reflective of systemic inflammation and explained the largest proportion of the total variance in a single component. Using the comparable NHANES III data (Age range: 30–65 y), a similar PCA analysis substituting serum ESR with serum fibrinogen replicated component loading magnitudes and percentage of variance explained. This ICS component score was also constructed previously [26].

Using refrigerated whole blood (5 ml) stored in EDTA buffer, ESR was tested within 24 h of blood draw using automated modified Westergren photochemical capillary stopped flow kinetic analysis. Based on the Mayo clinic reports, ESR’s reference range is 0–22 mm/hr for men and 0–29 mm/hr for women (https://www.mayoclinic.org/tests-procedures/sed-rate/about/pac-20384797). Similarly, hsCRP was analyzed with an immunoturbidimeter (Siemens/Behring Nephelometer II), utilizing 0.5–1 ml of plasma. A range of 1–3 mg/dL suggests average and 3–10 mg/dL high cardiovascular risk while hsCRP > 10 mg/dL reflects putative infection or chronic inflammation. Moreover, after collecting 0.5–1 ml sample of plasma prepared with heparin and refrigerated for < 30 days, albumin was measured using spectrophotometry (expected reference range: 3.6–5.1 g/dL). Finally, 0.5–1 ml of fasting serum was collected for iron determination, which was transported at room temperature (with heparin added), followed by refrigeration or freezing. Serum iron was determined with spectrophotometry, and reference ranges are age and sex-specific, [Men aged ≥ 30 y: 50–180 µg/dL; Women aged 20–49 y (40–190 µg /dL) and women aged 50 + y (45–160 µg/dL)].

### Cytokines

All available cytokines were used in our present study. Blood sample aliquots were frozen at −80 °C and stored for later use. Interleukin (IL) cytokines were assayed in one batch by Aushon (https://www.aushon.com/) Ciraplex® ULTRA Ultrasensitive Assays in femtogram/ml (fg/ml) detection levels. The serum samples were collected from HANDLS participants for studies of DNA repair and age-related microRNA changes [27, 28]. Cytokines selected for this study included IL-1β, IL-6, IL-10, IL-12, and IL-18 (in pg/ml), as they were previously linked with depressive symptoms [1, 2, 5].

### Covariates

#### Sociodemographic, lifestyle, and health-related potential confounders

All models were adjusted for sociodemographic factors, namely age, sex, race (White *vs*. AA), educational attainment categories (0 ≤ High School (HS); 1 = HS and 2 ≥ HS) and poverty status (below *vs*. above 125% the federal poverty line). Poverty status was categorized as such by using the US Census Bureau poverty thresholds for 2004 [29] relying on income, and total family size including children under age 18 years. Moreover, all models with the exception of those with cytokines were further adjusted for measured body mass index (kg/m^2^), current use of drugs (“opiates, marijuana or cocaine” = 1 *vs*. not = 0), and current smoking status (0: “never or former smoker” *vs*. 1 “current smoker”) without examining exposure-covariate associations. Those models were also adjusted for first-visit self-reported history of type 2 diabetes, hypertension, dyslipidemia, cardiovascular disease (stroke, congestive heart failure, non-fatal myocardial infarction or atrial fibrillation), inflammatory disease (multiple sclerosis, systemic lupus, gout, rheumatoid arthritis, psoriasis, thyroid disorder and Crohn’s disease) and use of NSAIDs (prescription and over-the-counter) over the past two weeks, as was done in previous studies [30, 31]. In models with cytokines, only sociodemographic covariates (age, sex, race, poverty status, education, employment status) and those that were deemed associated with the cytokines in a separate bivariate linear regression model were included.

#### Dietary potential confounders

Potential dietary confounders were considered in all models, being previously linked to reduced risk for depression, namely vitamins B-6, folate and B-12, total carotenoids (α-carotene, β-carotene, β-cryptoxanthin, lutein + zeaxanthin, lycopene), vitamin C and α-tocopherol [32–40] (expressed per 1,000 kcal) and the ratio of *n*−3 polyunsaturated fatty acids (PUFA):*n*-6 PUFA [41]. To emulate a multivariable nutrient density model, energy intake was entered as a covariate [42]. A measure of overall dietary quality, the Healthy Eating Index (HEI-2010) total score, (http://appliedresearch.cancer.gov/tools/hei/tools.html and http://handls.nih.gov/06Coll-dataDoc.htm) was also considered. Those covariates were included only with cytokines if they were associated with those exposures (OSM 1).

### Statistical analysis

All analyses were conducted using Stata 15.0 (StataCorp, College Station, TX) [43]. Baseline characteristics, including covariates and exposures, were compared across sex, race and by EDS status (CES-D score ≥ 16 *vs*. < 16, based on mean score across waves), using t-tests and ANOVA for continuous variables and *χ*^2^ tests for categorical variables. Second, several linear mixed-effects regression models on continuous CES-D total or on domain-specific score(s) were conducted to test associations with systemic markers of inflammation, including the ICS and cytokines, controlling for potential confounders. The time metric used was *TIME* elapsed since baseline visit (i.e. visit 1) in years. Visit 1 *TIME* was set at zero while Visit 2 *TIME* was set at the number of years elapsed, using age difference between visits 1 and 2. Cross-sectional relationships between baseline inflammation and baseline depressive symptoms were estimated using the main fixed effects of each inflammatory marker on the outcome of interest (γ_01a_ for π_0i_). Longitudinal relationships between baseline inflammation and annual rate of change in depressive symptoms (Δ) was estimated using the interaction fixed effects between *TIME* and each of those inflammatory markers (γ_11a_ for π_1i_). The methodology used is outline in OSM 2 [44–46]. Sex- and race-specific associations were tested by adding interaction terms to the multivariable mixed-effects regression models and stratifying by sex and race, separately. Third, a series of parametric survival models, assuming a Weibull baseline hazard function form, examined the association between baseline ICS and incident EDS, stratifying by sex and race [47]. Hazard ratios with 95% CIs were estimated for each SD increase in ICS in relation to instantaneous adjusted hazard of incident EDS, defined as a transition into a total CES-D score from < 16 to ≥ 16 or higher over time. Cox proportional hazards models were also carried out as a sensitivity analysis [47].

Non-random selection of participants from the initial probability HANDLS sample (*n* = 3,720) may cause bias due to systematic differentials in basic characteristics such as age, sex, race and socio-economic status between the final analytic sample and the excluded sample. A 2-stage Heckman process accounted for selection bias in all final models. At the first stage, a probit model with a binary outcome variable coded as selected = 1 *vs*. unselected = 0 was conducted from which an inverse mills ratio (derived from the predicted probability of being selected, conditional on the covariates baseline age, sex, race, poverty status and education) was estimated. At a second stage, this inverse mills ratio was entered into each mixed-effects regression model as a covariate, as was done in prior studies [48]. Two inverse mills ratio were computed, one for the ICS sub-sample and one for the cytokine sub-sample.

A type I error of 0.05 was used, with 0.05 < *p*-values < 0.10 judged as borderline significant for main effects, while *p*-value < 0.10 was considered significant for interaction terms [49] before family-wise Bonferroni correction for multiple testing [50], assuming CES-D_total_ and sub-domain scores are distinctive outcomes, while the 10 exposures that are conceptually related. This approach was adopted in several previous studies [46, 51]. Accounting for 10 exposures, type I error was reduced to 0.05/10 = 0.005 for main effects and 0.10/10 = 0.010 for interaction terms for the mixed-effects regression models, with no adjustment done when ICS was tested in the parametric survival models.

## Results

Table 1 displays study sample characteristics across sex, race and EDS status. Notably, mean CES-D was higher among women, as were means of hsCRP and ESR. The reverse was found for serum albumin and iron leading to a higher ICS among women. Similarly, AA and EDS^+^ participants had higher mean ICS compared with Whites and EDS^−^ individuals, respectively. Within the smaller sub-sample having cytokine data, a higher mean IL-6 was detected among EDS^+^ compared to EDS^−^ individuals. Sex differences in EDS status was also noted, whereby 46.7% of EDS^−^ were men (*vs*. 36.0% of EDS^+^), suggesting a higher prevalence among women. Educational attainment beyond high school was more prevalent among Whites and EDS^−^ individuals, compared with AA and EDS^+^ participants. A greater prevalence of employment and “above poverty” status was also observed among men, Whites and EDS^−^ individuals. Men were more likely current smokers and drug users, as were EDS^+^ individuals compared to women and EDS^−^ counterparts, respectively. Illicit drug use was also more prevalent among AA compared to Whites. Mean BMI and prevalence of hypertension and inflammatory conditions were higher among women, while among AA, hypertension and cardiovascular disease were more prevalent compared with Whites, with a reverse pattern by race observed for dyslipidemia. Similarly, EDS^+^ individuals had a higher prevalence of type 2 diabetes, hypertension, cardiovascular disease and inflammatory conditions. While HEI-2010 indicated better overall dietary quality in women and EDS^−^ individuals, other micronutrients exhibited different patterns across sex, race and EDS status. Most notably, total carotenoids, vitamins E, B-6 and folate (per 1,000 kcal) were higher among EDS^−^ individuals compared to the EDS^+^ groups. Folate intake per 1,000 kcal was also higher among Whites but the reverse pattern by race was true for vitamin B-12 and C intakes per 1000 kcal.

**Table 1:** Characteristics of HANDLS study participants by sex, race and EDS status [based on CES-D score (mean across waves)] ^a^

	Sex		*P*^*b*^	Race		*P*^*b*^	EDS status		*P*^*b*^
	Men	Women	Men *vs.* women	Whites	African-Americans	Whites *vs.* African-Americans	EDS^−^	EDS^+^	EDS^−^ *vs.* EDS^+^
%									
Depressive Symptoms	(*n* = 776)	(*n* = 988)		(*n* = 764)	(*n* = 1,000)		(*n* = 1,024)	(*n* = 740)	
CES-D, *Mean* ± *SEM*	14.1 ± 0.3	16.3 ± 0.3	**<** **0**.**001**	15.5 ± 0.4	15.1 ± 0.3	0.40	8.1 ± 0.1	25.3 ± 0.3	**<** **0**.**001**
Systemic inflammation markers	(*n* = 776)	(*n* = 988)		(*n* = 764)	(*n* = 1,000)		(*n* = 1,024)	(*n* = 740)	
High sensitivity C-reactive protein, mg/dL	4.2 ± 0.4	5.7 ± 0.3	**0**.**003**	4.4 ± 0.2	5.5 ± 0.4	**0**.**035**	4.6 ± 0.3	5.7 ± 0.5	**0**.**027**
Erythrocyte sedimentation rate, ESR	12.0 ± 0.5	20.0 ± 0.5	**<** **0**.**001**	13.1 ± 0.5	19.0 ± 0.6	**<** **0**.**001**	15.5 ± 0.5	17.6 ± 0.6	**0**.**008**
Serum albumin	4.35 ± 0.01	4.23 ± 0.01	**<** **0**.**001**	4.35 ± 0.01	4.24 ± 0.01	**<** **0**.**001**	4.30 ± 0.01	4.26 ± 0.01	**0**.**004**
Serum iron	93.3 ± 1.4	77.5 ± 1.2	**<** **0**.**001**	90.0 ± 1.4	80.1 ± 1.2	**<** **0**.**001**	86.7 ± 1.2	81.4 ± 1.4	**0**.**004**
Inflammation composite score, *z*-score	−0.40 ± 0.05	+ 0.31 ± 0.04	**<** **0**.**001**	−0.30 ± 0.04	+ 0.23 ± 0.04	**<** **0**.**001**	−0.11 ± 0.04	+ 0.15 ± 0.05	**<** **0**.**001**
Cytokines, pg/ml	(*n* = 52)	(*n* = 98)		(*n* = 37)	(*n* = 113)		(*n* = 79)	(*n* = 71)	
IL-1β	1.77 ± 0.38	1.72 ± 0.27	0.91	1.62 ± 0.52	1.77 ± 0.24	0.76	1.46 ± 0.25	2.03 ± 0.36	0.19
IL-6	16.7 ± 2.7	13.1 ± 1.3	0.19	14.0 ± 2.6	14.5 ± 1.5	0.86	11.4 ± 1.3	17.7 ± 2.2	**0**.**014**
IL-10	3.1 ± 0.8	2.3 ± 0.3	0.24	3.5 ± 0.7	2.3 ± 0.4	0.13	2.26 ± 0.34	2.98 ± 0.58	0.27
IL-12	1.52 ± 0.56	0.86 ± 0.14	0.15	1.3 ± 0.4	1.0 ± 0.3	0.61	0.89 ± 0.18	1.30 ± 0.41	0.35
IL-18	156.9 ± 14.6	174.2 ± 18.8	0.54	147.5 ± 12.8	174.9 ± 17.1	0.37	146.6 ± 9.2	192.3 ± 25.9	***0.084***
Sociodemographic characteristics	(*n* = 776)	(*n* = 988)		(*n* = 764)	(*n* = 1,000)		(*n* = 1,024)	(*n* = 740)	
Age (y), *Mean* *±* *SEM*	48.6 ± 0.3	48.7 ± 0.3	0.90	48.9 ± 0.3	48.5 ± 0.3	0.42	49.0 ± 0.3	48.2 ± 0.3	***0.08***
Sex, % men	__	__		43.2	44.6	0.55	46.7	36.0	**<** **0**.**001**
African-American, *%*	55.7	56.1	0.56	__	__		56.5	56.9	0.89
Education, *%*			0.29			**<** **0**.**001**			**<** **0**.**001**
<HS	7.7	5.9		9.2	4.8		5.8	8.0	
HS	58.3	57.7		52.1	62.4		52.9	64.9	
>HS	34.0	36.3		38.7	32.7		41.3	27.0	
Missing	0.0	0.1		0.0	0.1		0.0	0.1	
PIR ≥ 125%, *%*	61.5	56.1	**0**.**022**	67.4	51.6	**<** **0**.**001**	64.8	49.6	**<** **0**.**001**
Employed, *%*			**0**.**022**						
Yes	48.6	43.0		44.5	46.2	**<** **0**.**001**	53.6	34.2	**<** **0**.**001**
Missing	19.9	19.3		27.5	13.5		18.9	20.5	
Lifestyle and health-related factors									
Current smoking status, *%*			**<** **0**.**001**			0.14			**<** **0**.**001**
Currently smoking	51.9	42.0		48.4	43.7		40.3	54.7	
Missng	0.6	1.2		1.0	0.9		0.8	1.2	
Current use of illicit drugs, *%*			**<** **0**.**001**			**<** **0**.**001**	45.3	49.9	0.17
Used any type	60.8	36.5		41.1	51.9		1.7	1.5	
Missing	1.4	1.7		1.4	1.7				
	(*n* = 776)	(*n* = 988)		(*n* = 764)	(*n* = 1,000)		(*n* = 1,024)	(*n* *=* 740)	
Body mass index, kg/m^2^; Mean ± SEM	28.1 ± 0.2	31.5 ± 0.3	**<** **0**.**001**	30.2 ± 0.3	29.8 ± 0.2	0.38	29.9 ± 0.2	30.1 ± 0.3	0.54
Co-morbid conditions and NSAIDs	(*n* = 776)	(*n* = 988)		(*n* = 764)	(*n* = 1,000)		(*n* = 1,024)	(*n* *=* 740)	
Diabetes, %	13.4	15.1	0.32	14.7	14.1	0.74	12.7	16.6	**0**.**020**
Hypertension, %	37.0	41.9	**0**.**036**	33.6	44.4	**<** **0**.**001**	36.4	44.3	**0**.**001**
Dyslipidemia, %	25.8	27.0	0.55	31.8	22.4	**<** **0**.**001**	24.9	28.7	***0.08***
Cardiovascular disease^d^, %	11.9	14.9	***0.07***	10.1	16.2	**<** **0**.**001**	11.5	16.4	**0**.**003**
Inflammatory conditions^e^, %	9.5	18.6	**<** **0**.**001**	14.8	14.5	0.86	12.7	17.3	**0**.**007**
NSAIDS^f^, %	21.3	20.8	0.79	21.3	20.7	0.75	20.4	21.8	0.49
Dietary factors, daily intakes	(*n* = 776)	(*n* = 988)		(*n* = 764)	(*n* = 1,000)		(*n* = 1,024)	(*n* *=* 740)	
Energy, *kcal*	2,324 ± 39	1,739 ± 24	**<** **0**.**001**	2,015 ± 33	1,981 ± 31	0.46	2,043 ± 30	1,931 ± 35	**0**.**015**
Total carotenoids, mg/1,000 kcal	3,698 ± 157	4,058 ± 144.8	***0.09***	4,099 ± 163	3,743 ± 140	0.10	4,163 ± 143	3,529 ± 158	**0**.**003**
Vitamin A, RE/1,000 kcal	316.6 ± 20.4	342.1 ± 18.6	0.36	306.1 ± 8.3	349.9 ± 23.4	0.12	329.7 ± 15.6	332.6 ± 24.7	0.92
Vitamin C, mg/1,000 kcal	36.3 ± 1.4	40.6 ± 1.4	**0**.**032**	33.9 ± 1.3	42.4 ± 1.4	**<** **0**.**001**	40.2 ± 1.2	36.6 ± 1.7	***0.07***
Vitamin E, mg/1,000 kcal	3.10 ± 0.06	3.45 ± 0.07	**0**.**0003**	3.4 ± 0.1	3.2 ± 0.1	***0.08***	3.44 ± 0.07	3.10 ± 0.07	**<** **0**.**001**
Vitamin B-6,mg/1,000 kcal	0.93 ± 0.02	0.91 ± 0.01	0.30	0.93 ± 0.02	0.90 ± 0.01	0.25	0.95 ± 0.02	0.87 ± 0.01	**<** **0**.**001**
Vitamin B-12, μg/1,000 kcal	3.28 ± 0.21	3.01 ± 0.19	0.35	2.8 ± 0.1	3.4 ± 0.2	**0**.**043**	3.1 ± 0.2	3.2 ± 0.2	0.79
Folate, μg/1,000 kcal	181.6 ± 3.6	186.7 ± 3.1	0.28	198.3 ± 3.7	173.8 ± 2.9	**<** **0**.**001**	188.5 ± 3.2	178.8 ± 3.4	**0**.**039**
*n*3 PUFA:*n*6 PUFA ratio^c^	0.114 ± 0.003	0.114 ± 0.002	0.89	0.116 ± 0.002	0.112 ± 0.002	0.27	0.116 ± 0.002	0.111 ± 0.001	0.12
Healthy Eating Index-2010	41.8 ± 0.4	43.4 ± 0.4	**0**.**004**	30.2 ± 0.3	29.8 ± 0.2	0.38	44.1±0.4	40.6±0.4	**<0**.**001**

*AA* arachidonic acid, *ALA* α-linolenic acid, *CES-D* Center for Epidemiologic Studies-Depression scale, *DHA* Docosahexaenoic acid, *DPA* docosapentaenoic acid, *EPA* eicosapentaenoic acid, *HANDLS* Healthy Aging in Neighborhoods of Diversity Across the Lifespan, *HDL-C* high-density lipoprotein-cholesterol, *HS* high school, *IL* interleukin, LA linoleic acid; *n3* omega-3, *n6* omega-6, *NSAIDS* non-steroidal anti-inflammatory drugs, *PIR* poverty income ratio, *PUFA* polyunsaturated fatty acids, *SEM* standard error of the mean, *TC* total cholesterol

^a^Values are percent or Mean ± SEM or % ± SE

^b^*P* value was based on independent samples *t*-test when row variable is continuous and *χ*^2^ test when row variable is categorical

^c^*n3* PUFA included DHA + EPA + *n3*DPA + ALA. *n6* PUFA included AA + LA

^d^Cardiovascular disease include self-reported stroke, congestive heart failure, non-fatal myocardial infarction or atrial fibrillation

^e^Inflammatory conditions include multiple sclerosis, systemic lupus, gout, rheumatoid arthritis, psoriasis, thyroid disorder and Crohn’s disease

^f^Non-steroidal anti-inflammatory drugs (NSAIDS) include over the counter and prescription drugs in that category

Bold values are for *P* < 0.05. Bold and italic values are for *P* < 0.10

**Fig. 1:**
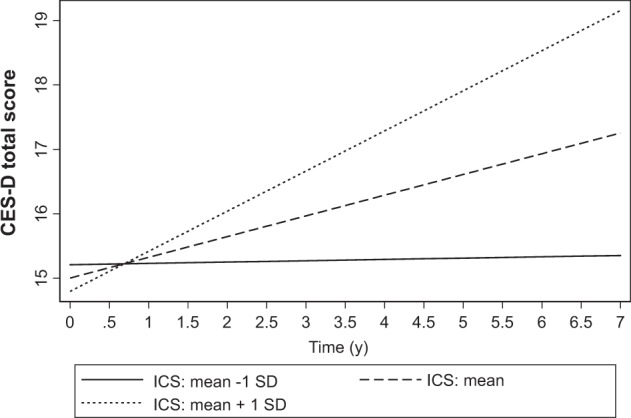
Predictive margins of CES-D total score by ICS among Whites from multiple linear mixed-effects regression model, HANDLS 2004–2013

Using mixed-effects linear regression models (OSM 2), we examined the associations of systemic inflammatory markers and cytokines with baseline and longitudinal annual rate of change in depressive symptoms (Δ), while stratifying our analyses by sex and race, separately. Table 2 focuses on the total CES-D score and Table 3 shows results for each of the 4 CES-D component scores. After correction for multiple testing, and among Whites only, hsCRP, ICS and IL-1β were all associated with a faster ΔCES-D_total_, with significant racial differences. The result for ICS among Whites is illustrated as predictive margins of CES-D_total_ across 1 SD changes in ICS in Fig. 1. This Figure shows clearly that the rate of change in CES-D_total_ over time is increased with each higher level of ICS among Whites. In contrast, IL-12 was directly associated with a faster ΔCES-D_total_ though only among men and AA, with significant sex and race differentials (Table 2). The race-specific associations of hsCRP, ICS, IL-1β and the sex-specific association of IL-12 with ΔCES-D trajectory were consistently detected for the “depressed affect” domain (component 2) (Table 3). For component 1 of CES-D, and among men only, lower serum albumin and higher ICS were both associated with a higher score suggesting greater “somatic complaints” at baseline. The third component of the CES-D (“positive affect”) was longitudinally associated with IL-10 among AA and IL-12 among men, suggesting that those cytokines were specifically linked to reducing positive affect over time in those population groups. Finally, the fourth component of the CES-D (“interpersonal problems”) was cross-sectionally associated with IL-6 among AA and IL-10 among Whites.

**Table 2:** Analysis of baseline systemic inflammatory markers, cytokines and longitudinal change in CES-D score (sex- and race-stratified), mixed-effects linear regression analysis, HANDLS study, 2004–2013

	Men		Women		Whites		African-Americans	
	γ ± SEE	*P*	γ ± SEE	*P*	γ ± SEE	*p*	γ ± SEE	*p*
High-sensitivity C-reactive protein, hsCRP	*N* = 776	*N*′ = 1,308	*N* = 991	*N*′ = 1,746	*N* = 764	*N*′ = 1,285	*N* = 1,003	*N*′ = 1,769
hsCRP (γ_011_ for π_0i_)	+ 0.038 ± 0.030	0.19	−0.020 ± 0.039	0.61	−0.091 ± 0.061	0.13	+ 0.024 ± 0.026	0.35
hsCRP × Time (γ_111_ for π_1i_)	−0.003 ± 0.008	0.66	+ 0.010 ± 0.009	0.22	**+** **0**.**045** **±** **0**.**016**^**c,e**^	**0**.**006**	−0.002 ± 0.006	0.71
Erythrocyte Sedimentation Rate, ESR	*N* = 776	*N*′ = 1,308	*N* = 991	*N*′ = 1,746	*N* = 764	*N*′ = 1,285	*N* = 1,003	*N*′ = 1,769
ESR (γ_012_ for π_0i_)	+ 0.020 ± 0.024	0.41	−0.017 ± 0.022	0.44	−0.023 ± 0.032	0.46	−0.003 ± 0.019	0.88
ESR × Time (γ_112_ for π_1i_)	−0.002 ± 0.006	0.72	+ 0.002 ± 0.005	0.63	+ 0.009 ± 0.009	0.32	+ 0.000 ± 0.004	0.95
Albumin, ALB	*N* = 776	*N*′ = 1,308	*N* = 991	*N*′ = 1,746	*N* = 764	*N*′ = 1,285	*N* = 1,003	*N*′ = 1,769
ALB (γ_013_ for π_0i_)	***−1***.***850*** ***±*** ***1***.***067***^**c**^	***0***.***083***	−1.312 ± 1.242	0.29	−1.387 ± 1.364	0.31	−1.174 ± 1.000	0.24
ALB × Time (γ_113_ for π_1i_)	+ 0.341 ± 0.248	0.17	−0.092 ± 0.289	0.75	−0.585 ± 0.364	0.11	+ 0.267 ± 0.222	0.23
Serum Iron, IRON	*N* = 776	*N*′ = 1,308	*N* = 991	*N*′ = 1,746	*N* = 764	*N*′ = 1,285	*N* = 1,003	*N*′ = 1,769
IRON (γ_014_ for π_0i_)	−0.005 ± 0.009	0.58	−0.008 ± 0.010	0.43	+ 0.006 ± 0.010	0.54	−0.012 ± 0.009	0.16
IRON × Time (γ_114_ for π_1i_)	−0.001 ± 0.002	0.66	−0.001 ± 0.002	0.52	**−0**.**006** **±** **0**.**002**^**c**^	**0**.**015**	+ 0.002 ± 0.002	0.19
Inflammation composite score, ICS	*N* = 776	*N*′ = 1,308	*N* = 991	*N*′ = 1,746	*N* = 764	*N*′ = 1,285	*N* = 1,003	*N*′ = 1,769
ICS (γ_015_ for π0i)	***+*** ***0***.***487*** ***±*** ***0***.***276***^***c***^	***0***.***078***	+ 0.045 ± 0.316	0.89	−0.207 ± 0.387	0.59	+ 0.301 ± 0.244	0.22
ICS × Time (γ_115_ for π1i)	−0.047 ± 0.067	0.48	+ 0.082 ± 0.073	0.26	**+** **0**.**301** **±** **0**.**099**^**c,e**^	**0**.**002**	−0.060 ± 0.057	0.29
IL-1β	*N* = 52	*N*′ = 90	*N* = 98	*N*′ = 167	*N* = 37	*N*′ = 62	*N* = 113	*N*′ = 195
IL-1β (γ_016_ for π0i)	+ 0.140 ± 0.566	0.81	+ 0.247 ± 0.376	0.51	+ 0.811 ± 0.618	0.19	+ 0.192 ± 0.357	0.59
IL-1β × Time (γ_116_ for π1i)	+ 0.280 ± 0.218	0.20	+ 0.043 ± 0.095	0.65	**+** **0**.**639** **±** **0**.**226**^**c,e**^	**0**.**005**	+ 0.004 ± 0.096	0.97
IL-6	*N* = 52	*N*′ = 90	*N* = 98	*N*′ = 167	*N* = 37	*N*′ = 62	*N* = 113	*N*′ = 195
IL-6 (γ_017_ for π0i)	+ 0.062 ± 0.076	0.41	***+*** ***0***.***138*** ***±*** ***0***.***078***^**c**^	***0***.***078***	***−0***.***180*** ***±*** ***0***.***101***^***c***^	***0***.***075***	**+** **0**.**177** **±** **0**.**058**^**c,d**^	**0**.**002**
IL-6 × Time (γ_117_ for π1i)	−0.012 ± 0.031	0.69	+ 0.005 ± 0.023	0.85	**+** **0**.**060** **±** **0**.**026**	**0**.**018**	−0.016 ± 0.023	0.48
IL-10	*N* = 52	*N*′ = 90	*N* = 98	*N*′ = 167	*N* = 37	*N*′ = 62	*N* = 113	*N*′ = 195
IL-10 (γ_018_ for π0i)	+ 0.069 ± 0.026	0.79	+ 0.158 ± 0.400	0.69	+ 0.044 ± 0.432	0.92	+ 0.198 ± 0.246	0.42
IL-10 × Time (γ_118_ for π1i)	+ 0.190 ± 0.178	0.29	−0.056 ± 0.095	0.55	−0.068 ± 0.098	0.49	+ 0.157 ± 0.165	0.34
IL-12	*N* = 52	*N*′ = 90	*N* = 98	*N*′ = 167	*N* = 37	*N*′ = 62	*N* = 113	*N*′ = 195
IL-12 (γ_019_ for π0i)	+ 0.022 ± 0.363	0.95	−0.354 ± 0.737	0.63	−0.524 ± 0.787	0.51	+ 0.118 ± 0.340	0.72
IL-12 × Time (γ_119_ for π1i)	**+** **0**.**662** **±** **0**.**219**^**c,e**^	**0**.**003**	+ 0.143 ± 0.181	0.43	+ 0.148 ± 0.194	0.45	**+** **0**.**572** **±** **0**.**212**^**c,e**^	**0**.**007**
IL-18	*N* = 52	*N*′ = 90	*N* = 98	*N*′ = 167	*N* = 37	*N*′ = 62	*N* = 113	*N*′ = 195
IL-18 (γ_020_ for π0i)	+ 0.002 ± 0.014	0.88	+ 0.004 ± 0.006	0.48	−0.016 ± 0.024	0.49	+ 0.004 ± 0.005	0.48
IL-18 × Time (γ_120_ for π1i)	−0.004 ± 0.005	0.34	−0.002 ± 0.003	0.52	**−0**.**017** **±** **0**.**008**^**c**^	**0**.**035**	+ 0.000 ± 0.003	0.97

*CES-D* Center for Epidemiologic Studies-Depression scale, *HANDLS* Healthy Aging in Neighborhoods of Diversity Across the Lifespan, *HS* high school, *IL* interleukin, *n3* omega-3, *n6* omega-6, *PUFA* polyunsaturated fatty acids, *SEE* standard error of the estimate

^a^Models were further adjusted for other covariates (main effects and interaction with time). Time at baseline visit was set to zero. Covariates considered as potential confounders included: baseline age was centered at 50 y, sex, race, PIR, education, employment status, total energy intake centered at 2000 kcal/d, total carotenoid intake at 3 mg/1,000 kcal/d, vitamin C intake at 30 mg/1,000 kcal/d, vitamin A intake at 300 RE/1,000 kcal/d, vitamin E at 3 mg/1,000 kcal/d, vitamin B-6 at 0.8 mg/1,000 kcal/d, vitamin B-12 at 3 μg/1,000 kcal/d, folate at 170 μg/1,000 kcal/d, n-3 PUFA:n-6 PUFA at 0.11. Healthy Eating Index-2010 was centered at 42, body mass index at 30, co-morbid conditions (diabetes, hypertension, dyslipidemia, CVD, inflammatory conditions) and use of NSAIDs. All these covariates were entered in models with ICS and individual component markers (i.e., hsCRP, ESR, ALBUMIN and IRON). In models with cytokines, only sociodemographic covariates (age, sex, race, PIR, education, employment status) and those that were deemed associated with the cytokines in a separate bivariate linear regression model were included. Models stratified by sex or race excluded those covariates in main and interaction effects with *TIME*

^b^*N* = number of participants in the analysis; *N*′ = total number of visits included in the analysis. Findings that were significant at a type I error of 0.05 are bolded

^c^In a separate model with interaction of inflammation marker/cytokine exposures by (sex/race) by TIME, including all other terms in the current model, *p* < 0.10 for null hypothesis that this interaction term is = 0

^d^*P* < 0.005 for exposure main effects

^e^*P* < 0.010 for interaction term (exposure × TIME)

**Table 3:** Analysis of baseline systemic inflammatory markers, cytokines and longitudinal change in CES-D component scores (sex- and race-stratified), mixed-effects linear regression analysis, HANDLS study, 2004–2013

	Men		Women		Whites		African-Americans	
	γ ± SEE	*P*	γ ± SEE	*P*	γ ± SEE	*P*	γ ± SEE	*p*
Y = CES-D component 1: Somatic complaints								
High-sensitivity C-reactive protein, hsCRP	*N* = 776	*N*′ = 1,312	*N* = 991	*N*′ = 1,752	*N* = 764	*N*′ = 1,289	*N* = 1,003	*N*′ = 1,776
hsCRP (γ_011_ for π_0i_)	**+** **0**.**030** **±** **0**.**011**^**c**^	**0**.**008**	−0.009 ± 0.015	0.55	−0.025 ± 0.023^**c**^	0.27	***+*** ***0***.***019*** ***±*** ***0***.***010***	***0***.***068***
hsCRP × Time (γ_111_ for π_1i_)	−0.005 ± 0.003	0.11	+ 0.004 ± 0.004	0.24	**+** **0**.**016** **±** **0**.**007**^**c**^	**0**.**014**	−0.003 ± 0.003	0.27
Erythrocyte sedimentation rate, ESR	*N* = 776	*N*′ = 1,312	*N* = 991	*N*′ = 1,753	*N* = 764	*N*′ = 1,289	*N* = 1,003	*N*′ = 1,776
ESR (γ_012_ for π_0i_)	+ 0.009 ± 0.009	0.36	+ 0.004 ± 0.009	0.61	−0.006 ± 0.012	0.65	+ 0.008 ± 0.007	0.31
ESR × Time (γ_112_ for π_1i_)	−0.001 ± 0.003	0.75	−0.001 ± 0.002	0.66	+ 0.004 ± 0.004	0.29	−0.002 ± 0.002	0.41
Albumin, ALB	*N* = 776	*N*′ = 1,312	*N* = 991	*N*′ = 1,753	*N* = 764	*N*′ = 1,289	*N* = 1,003	*N*′ = 1,776
ALB (γ_013_ for π_0i_)	**−1**.**192** **±** **0**.**417**^**c,d**^	**0**.**004**	**−1**.**152** **±** **0**.**487**	**0**.**016**	**−1**.**238** **±** **0**.**515**^**c**^	**0**.**016**	**−0**.**913** **±** **0**.**398**	**0**.**022**
ALB × Time (γ_113_ for π_1i_)	+ 0.146 ± 0.105	0.17	+ 0.074 ± 0.121	0.54	−0.076 ± 0.148	0.61	+ 0.122 ± 0.095	0.20
Serum Iron, IRON	*N* = 776	*N*′ = 1,312	*N* = 991	*N*′ = 1,753	*N* = 764	*N*′ = 1,289	*N* = 1,003	*N*′ = 1,776
IRON (γ_014_ for π_0i_)	−0.005 ± 0.004	0.17	−0.005 ± 0.004	0.15	−0.001 ± 0.004^**c**^	0.71	***−0***.***007*** ***±*** ***0***.***004***	***0***.***059***
IRON × Time (γ_114_ for π_1i_)	+ 0.000 ± 0.001	0.92	+ 0.000 ± 0.001	0.85	***−0***.***002*** ***±*** ***0***.***010***^c^	***0***.***062***	***+*** ***0***.***0014*** ***±*** ***0***.***0008***	***0***.***072***
Inflammation Composite Score, ICS	*N* = 776	*N*′ = 1,312	*N* = 991	*N*′ = 1,753	*N* = 764	*N*′ = 1,289	*N* = 1,003	*N*′ = 1,776
ICS (γ_015_ for π0i)	**+** **0**.**336** **±** **0**.**108**	**0**.**002**^**c,d**^	+ 0.184 ± 0.122	0.13	+ 0.108 ± 0.146^**c**^	0.46	**+** **0**.**267** **±** **0**.**097**	**0**.**006**
ICS × Time (γ_115_ for π1i)	−0.038 ± 0.028	0.17	−0.002 ± 0.031	0.95	**+** **0**.**090** **±** **0**.**040**^**c**^	**0**.**026**	***−0***.***047*** ***±*** ***0***.***024***	***0***.***052***
IL-1β	*N* = 52	*N*′ = 90	*N* = 98	*N*′ = 167	*N* = 37	*N*′ = 37	*N* = 113	*N*′ = 195
IL-1β (γ_016_ for π0i)	+ 0.113 ± 0.295	0.70	−0.033 ± 0.147	0.82	+ 0.384 ± 0.243	0.11	−0.016 ± 0.139	0.91
IL-1β × Time (γ_116_ for π1i)	+ 0.008 ± 0.079	0.92	+ 0.006 ± 0.034	0.87	***+*** ***0***.***174*** ***±*** ***0***.***092***^***c***^	***0***.***059***	−0.017 ± 0.033	0.61
IL-6	*N* = 52	*N*′ = 90	*N* = 98	*N*′ = 167	*N* = 37	*N*′ = 62	*N* = 113	*N*′ = 195
IL-6 (γ_017_ for π0i)	+ 0.037 ± 0.029	0.21	+ 0.037 ± 0.031	0.23	−0.047 ± 0.041^**c**^	0.26	**+** **0**.**060** **±** **0**.**023**	**0**.**010**
IL-6 × Time (γ_117_ for π1i)	+ 0.009 ± 0.011	0.43	+ 0.000 ± 0.009	0.99	**+** **0**.**025** **±** **0**.**013**^**c**^	**0**.**039**	−0.002 ± 0.008	0.79
IL-10	*N* = 52	*N*′ = 90	*N* = 98	*N*′ = 167	*N* = 37	*N*′ = 62	*N* = 113	*N*′ = 195
IL-10 (γ_018_ for π0i)	+ 0.076 ± 0.104	0.47	−0.075 ± 0.156	0.63	−0.029 ± 0.176	0.86	+ 0.078 ± 0.097	0.42
IL-10 × Time (γ_118_ for π1i)	+ 0.054 ± 0.064	0.40	−0.017 ± 0.034	0.62	+ 0.000 ± 0.936	0.99	−0.003 ± 0.059	0.96
IL-12	*N* = 52	*N*′ = 90	*N* = 98	*N*′ = 167	*N* = 37	*N*′ = 62	*N* = 113	*N*′ = 195
IL-12 (γ_019_ for π0i)	+ 0.082 ± 0.146	0.58	−0.259 ± 0.288	0.37	−0.167 ± 0.328	0.61	+ 0.077 ± 0.135	0.57
IL-12 × Time (γ_119_ for π1i)	**+** **0**.**171** **±** **0**.**083**^**c**^	**0**.**039**	+ 0.028 ± 0.064	0.66	+ 0.074 ± 0.070	0.29	+ 0.079 ± 0.079	0.33
IL-18	*N* = 52	*N*′ = 90	*N* = 98	*N*′ = 167	*N* = 37	*N*′ = 62	*N* = 113	*N*′ = 195
IL-18 (γ_020_ for π0i)	+ 0.004 ± 0.005	0.48	+ 0.002 ± 0.002	0.44	−0.000 ± 0.002	0.79	+ 0.001 ± + 0.002	0.52
IL-18 × Time (γ_120_ for π1i)	−0.000 ± 0.002	0.95	−0.001 ± 0.001	0.37	+ 0.001 ± 0.001	0.36	−0.000 ± 0.001	0.70
Y **=** CES-D component 2: Depressed affect								
High-sensitivity C-reactive protein, hsCRP	*N* = 776	*N*′ = 1,312	*N* = 991	*N*′ = 1,752	*N* = 764	*N*′ = 1,292	*N* = 1,003	*N*′ = 1,772
hsCRP (γ_011_ for π_0i_)	+ 0.009 ± 0.013	0.48	−0.011 ± 0.017	0.51	−0.038 ± 0.026	0.15	+ 0.003 ± 0.0011	0.81
hsCRP × Time (γ_111_ for π_1i_)	−0.0003 ± 0.0035	0.93	+ 0.005 ± 0.004	0.18	**+** **0**.**020** **±** **0**.**007**^**c,e**^	**0**.**007**	+ 0.000 ± 0.003	0.97
Erythrocyte Sedimentation Rate, ESR	*N* = 776	*N*′ = 1,312	*N* = 991	*N*′ = 1,752	*N* = 764	*N*′ = 1,292	*N* = 1,003	*N*′ = 1,772
ESR (γ_012_ for π_0i_)	+ 0.008 ± 0.010	0.43	−0.015 ± 0.010	0.13	−0.018 ± 0.014	0.19	−0.005 ± 0.008	0.57
ESR × Time (γ_112_ for π_1i_)	−0.003 ± 0.003	0.35	+ 0.003 ± 0.002	0.18	+ 0.004 ± 0.004	0.31	+ 0.001 ± 0.002	0.66
Albumin, ALB	*N* = 776	*N*′ = 1,312	*N* = 991	*N*′ = 1,752	*N* = 764	*N*′ = 1,292	*N* = 1,003	*N*′ = 1,772
ALB (γ_013_ for π_0i_)	−0.407 ± 0.461	0.38	+ 0.013 ± 0.550	0.98	−0.036 ± 0.594	0.951	−0.208 ± 0.443	0.64
ALB × Time (γ_113_ for π_1i_)	+ 0.160 ± 0.116	0.17	−0.096 ± 0.132	0.47	**−0**.**348** **±** **0**.**161**^**c**^	**0**.**031**	+ 0.165 ± 0.105	0.12
Serum Iron, IRON	*N* = 776	*N*′ = 1,312	*N* = 991	*N*′ = 1,752	*N* = 764	*N*′ = 1,292	*N* = 1,003	*N*′ = 1,772
IRON (γ_014_ for π_0i_)	**−0**.**202** **±** **0**.**093**^**c**^	**0**.**030**	+ 0.000 ± 0.004	0.96	+ 0.005 ± 0.004	0.24	−0.003 ± 0.004	0.52
IRON × Time (γ_114_ for π_1i_)	−0.000 ± 0.004	0.95	−0.001 ± 0.001	0.27	**−0**.**0023** **±** **0**.**0010**^**c**^	**0**.**028**	+ 0.0007 ± 0.0009	0.44
Inflammation Composite Score, ICS	*N* = 776	*N*′ = 1,312	*N* = 991	*N*′ = 1,752	*N* = 764	*N*′ = 1,292	*N* = 1,003	*N*′ = 1,772
ICS (γ_015_ for π0i)	+ 0.118 ± 0.119	0.32	−0.142 ± 0.140	0.31	−0.240 ± 0.168	0.15	+ 0.025 ± 0.109	0.82
ICS × Time (γ_115_ for π1i)	−0.033 ± 0.031	0.29	+ 0.063 ± 0.033	0.06^**c**^	**+** **0**.**139** **±** **0**.**044**^**c,e**^	**0**.**002**	−0.018 ± 0.027	0.49
IL-1β	*N* = 52	*N*′ = 90	*N* = 98	*N*′ = 168	*N* = 37	*N*′ = 63	*N* = 113	*N*′ = 195
IL-1β (γ_016_ for π0i)	−0.044 ± 0.241	0.86	+ 0.107 ± 0.194	0.58	+ 0.153 ± 0.284	0.59	+ 0.088 ± 0.177	0.62
IL-1β × Time (γ_116_ for π1i)	***+*** ***0***.***180*** ***±*** ***0***.***092***^**c**^	***0***.***050***	+ 0.042 ± 0.052	0.42	**+** **0**.**373** **±** **0**.**123**^**c,e**^	**0**.**003**	+ 0.014 ± 0.048	0.77
IL-6	*N* = 52	*N*′ = 90	*N* = 98	*N*′ = 168	*N* = 37	*N*′ = 63	*N* = 113	*N*′ = 195
IL-6 (γ_017_ for π0i)	+ 0.011 ± 0.032	0.72	**+** **0**.**092** **±** **0**.**039**^**c**^	**0**.**020**	−0.044 ± 0.047	0.35	+ 0.076 ± 0.029	0.009^**c**^
IL-6 × Time (γ_117_ for π1i)	−0.012 ± 0.014	0.37	−0.002 ± 0.013	0.87	+ 0.015 ± 0.017	0.37	−0.011 ± 0.011	0.32
IL-10	*N* = 52	*N*′ = 90	*N* = 98	*N*′ = 168	*N* = 37	*N*′ = 63	*N* = 113	*N*′ = 195
IL-10 (γ_018_ for π0i)	−0.022 ± 0.112	0.84	+ 0.103 ± 0.207	0.62	−0.038 ± 0.203	0.85	+ 0.052 ± 0.122	0.67
IL-10 × Time (γ_118_ for π1i)	+ 0.042 ± 0.074	0.57	−0.035 ± 0.053	0.50	−0.028 ± 0.053	0.59	+ 0.053 ± 0.078	0.50
IL-12	*N* = 52	*N*′ = 90	*N* = 98	*N*′ = 168	*N* = 37	*N*′ = 63	*N* = 113	*N*′ = 195
IL-12 (γ_019_ for π0i)	−0.064 ± 0.154	0.68	−0.098 ± 0.370	0.64	−0.284 ± 0.372	0.45	−0.012 ± 0.167	0.94
IL-12 × Time (γ_119_ for π1i)	**+** **0**.**260** **±** **0**.**093**^**c,e**^	**0**.**005**	+ 0.084 ± 0.098	0.40	+ 0.075 ± 0.102^**c**^	0.46	**+** **0**.**247** **±** **0**.**101**	**0**.**015**
IL-18	*N* = 52	*N*′ = 90	*N* = 98	*N*′ = 168	*N* = 37	*N*′ = 62	*N* = 113	*N*′ = 195
IL-18 (γ_020_ for π0i)	+ 0.002 ± 0.006	0.76	+ 0.003 ± 0.003	0.32	−0.007 ± 0.010	0.51	+ 0.003 ± 0.003	0.25
IL-18 × Time (γ_120_ for π1i)	−0.003 ± 0.002	0.16	−0.000 ± 0.002	0.77	***−0***.***008*** ***±*** ***0***.***004***^***c***^	***0***.***056***	−0.000 ± 0.001	0.99
Y **=** CES-D component 3: Positive affect								
High-sensitivity C-reactive protein, hsCRP	*N* = 776	*N*′ = 1,310	*N* = 991	*N*′ = 1,754	*N* = 764	*N*′ = 1,291	*N* = 1,003	*N*′ = 1,773
hsCRP (γ_011_ for π_0i_)	+ 0.001 ± 0.007	0.90	−0.005 ± 0.009	0.61	+ 0.011 ± 0.015	0.45	−0.003 ± 0.006	0.64
hsCRP × Time (γ_111_ for π_1i_)	−0.003 ± 0.002	0.23	−0.000 ± 0.002	0.89	−0.003 ± 0.004	0.43	−0.001 ± 0.002	0.47
Erythrocyte Sedimentation Rate, ESR	*N* = 776	*N*′ = 1,310	*N* = 991	*N*′ = 1,754	*N* = 764	*N*′ = 1,291	*N* = 1,003	*N*′ = 1,773
ESR (γ_012_ for π_0i_)	−0.007 ± 0.006	0.24	+ 0.005 ± 0.005	0.33	−0.005 ± 0.008	0.56	+ 0.003 ± 0.004	0.47
ESR × Time (γ_112_ for π_1i_)	+ 0.001 ± 0.002	0.46	−0.001 ± 0.001	0.64	+ 0.001 ± 0.002	0.75	−0.000 ± 0.001	0.91
Albumin, ALB	*N* = 776	*N*′ = 1,310	*N* = 991	*N*′ = 1,754	*N* = 764	*N*′ = 1,291	*N* = 1,003	*N*′ = 1,773
ALB (γ_013_ for π_0i_)	+ 0.136 ± 0.261	0.60	+ 0.109 ± 0.299	0.72	+ 0.189 ± 0.344	0.58	−0.012 ± 0.239	0.96
ALB × Time (γ_113_ for π_1i_)	−0.037 ± 0.071	0.61	+ 0.020 ± 0.072	0.78	+ 0.058 ± 0.092	0.53	−0.001 ± 0.060	0.98
Serum Iron, IRON	*N* = 776	*N*′ = 1,310	*N* = 991	*N*′ = 1,754	*N* = 764	*N*′ = 1,291	*N* = 1,003	*N*′ = 1,773
IRON (γ_014_ for π_0i_)	−0.001 ± 0.002	0.75	+ 0.001 ± 0.002	0.57	−0.0015 ± 0.0020	0.54	+ 0.001 ± 0.002	0.51
IRON × Time (γ_114_ for π_1i_)	**+** **0**.**0013** **±** **0**.**0006**^**c**^	**0**.**019**	+ 0.000 ± 0.001	0.63	***+*** ***0***.***0011*** ***±*** ***0***.***0006***^**c**^	***0***.***069***	+ 0.0001 ± 0.0004	0.80
Inflammation Composite Score, ICS	*N* = 776	*N*′ = 1,310	*N* = 991	*N*′ = 1,754	*N* = 764	*N*′ = 1,291	*N* = 1,003	*N*′ = 1,773
ICS (γ_015_ for π0i)	−0.034 ± 0.068	0.62	−0.005 ± 0.076	0.95	+ 0.000 ± 0.097	0.99	−0.002 ± 0.059	0.96
ICS × Time (γ_115_ for π1i)	−0.018 ± 0.019	0.37	−0.011 ± 0.018	0.53	−0.030 ± 0.025	0.22	−0.007 ± 0.015	0.65
IL-1β	*N* = 52	*N*′ = 90	*N* = 98	*N*′ = 168	*N* = 37	*N*′ = 63	*N* = 113	*N*′ = 195
IL-1β (γ_016_ for π0i)	−0.017 ± 0.119	0.89	−0.072 ± 0.088	0.41	−0.042 ± 0.146^c^	0.78	−0.088 ± 0.082	0.29
IL-1β × Time (γ_116_ for π1i)	−0.070 ± 0.056	0.21	−0.014 ± 0.025	0.58	**−0**.**106** **±** **0**.**054**^**c**^	**0**.**048**	−0.001 ± 0.024	0.95
IL-6	*N* = 52	*N*′ = 90	*N* = 98	*N*′ = 168	*N* = 37	*N*′ = 63	*N* = 113	*N*′ = 195
IL-6 (γ_017_ for π0i)	−0.006 ± 0.015	0.69	+ 0.0015 ± 0.018	0.93	***+*** ***0***.***044*** ***±*** ***0***.***024***^c^	***0***.***064***	−0.020 ± 0.014	0.14
IL-6 × Time (γ_117_ for π1i)	+ 0.002 ± 0.008	0.83	−0.004 ± 0.006	0.49	−0.003 ± 0.006	0.67	−0.002 ± 0.006	0.72
IL-10	*N* = 52	*N*′ = 90	*N* = 98	*N*′ = 168	*N* = 37	*N*′ = 63	N = 113	*N*′ = 195
IL-10 (γ_018_ for π0i)	+ 0.014 ± 0.054	0.79	+ 0.065 ± 0.094	0.49	+ 0.099 ± 0.095	0.30	−0.011 ± 0.056	0.85
IL-10 × Time (γ_118_ for π1i)	**−0**.**104** **±** **0**.**043**^**c**^	**0**.**017**	−0.008 ± 0.026	0.76	+ 0.006 ± 0.024	0.81	**−0**.**106** **±** **0**.**037**^**e**^	**0**.**005**
IL-12	*N* = 52	*N*′ = 90	*N* = 98	*N*′ = 168	*N* = 37	*N*′ = 63	*N* = 113	*N*′ = 195
IL-12 (γ_019_ for π0i)	+ 0.041 ± 0.077	0.59	+ 0.128 ± 0.173	0.46	+ 0.229 ± 0.159	0.15	+ 0.007 ± 0.078	0.93
IL-12 × Time (γ_119_ for π1i)	**−0**.**200** **±** **0**.**050**^**c,e**^	**<** **0**.**001**	−0.038 ± 0.048	0.44	+ 0.008 ± 0.042^c^	0.85	**−0**.**200** **±** **0**.**047**^**e**^	**<** **0**.**001**
IL-18	*N* = 52	*N*′ = 90	*N* = 98	*N*′ = 168	*N* = 37	*N*′ = 63	*N* = 113	*N*′ = 195
IL-18 (γ_020_ for π0i)	+ 0.003 ± 0.003	0.27	+ 0.001 ± 0.001	0.37	+ 0.008 ± 0.006	0.20	+ 0.001 ± 0.001	0.31
IL-18 × Time (γ_120_ for π1i)	+ 0.001 ± 0.001	0.29	−0.000 ± 0.001	0.91	**+** **0**.**004** **±** **0**.**002**^**c**^	**0**.**026**	−0.000 ± 0.001	0.59
Y **=** CES-D component 4: Interpersonal problems								
High-sensitivity C-reactive protein, hsCRP	*N* = 776	*N*′ = 1,313	*N* = 991	*N*′ = 1,755	*N* = 764	*N*′ = 1,292	*N* = 1,003	*N*′ = 1,776
hsCRP (γ_011_ for π_0i_)	−0.000 ± 0.004	0.99	−0.005 ± 0.005	0.31	-**0**.**014** **±** **0**.**007**^**c**^	**0**.**046**	−0.000 ± 0.003	0.91
hsCRP × Time (γ_111_ for π_1i_)	−0.001 ± 0.001	0.54	+ 0.001 ± 0.001	0.53	**+** **0**.**005** **±** **0**.**002**^**c**^	**0**.**014**	−0.001 ± 0.001	0.41
Erythrocyte Sedimentation Rate, ESR	*N* = 776	*N*′ = 1,313	*N* = 991	*N*′ = 1,755	*N* = 764	*N*′ = 1,292	*N* = 1,003	*N*′ = 1,776
ESR (γ_012_ for π_0i_)	−0.004 ± 0.003	0.24	−0.001 ± 0.003	0.68	−0.004 ± 0.004	0.36	−0.002 ± 0.003	0.36
ESR × Time (γ_112_ for π_1i_)	**+** **0**.**002** **±** **0**.**001**^**c**^	**0**.**030**	−0.000 ± 0.001	0.64	+ 0.002 ± 0.0012	0.17	+ 0.000 ± 0.001	0.80
Albumin, ALB	*N* = 776	*N*′ = 1,313	*N* = 991	*N*′ = 1,755	*N* = 764	*N*′ = 1,292	*N* = 1,003	*N*′ = 1,776
ALB (γ_013_ for π_0i_)	−0.116 ± 0.151	0.44	−0.098 ± 0.146	0.50	−0.001 ± 0.164	1.00	−0.077 ± 0.135	0.56
ALB × Time (γ_113_ for π_1i_)	+ 0.020 ± 0.042	0.64	−0.034 ± 0.040	0.39	***−0***.***086*** ***±*** ***0***.***050***^**c**^	***0***.***088***	+ 0.019 ± 0.035	0.60
Serum Iron, IRON	*N* = 776	*N*′ = 1,313	*N* = 991	*N*′ = 1,755	*N* = 764	*N*′ = 1,292	*N* = 1,003	*N*′ = 1,776
IRON (γ_014_ for π_0i_)	−0.001 ± 0.001	0.51	−0.001 ± 0.001	0.33	+ 0.001 ± 0.001	0.52	−0.0018 ± 0.0012	0.14
IRON × Time (γ_114_ for π_1i_)	***+*** ***0***.***0006*** ***±*** ***0***.***0003***^**c**^	***0***.***081***	−0.000 ± 0.000	0.42	−0.0002 ± 0.0003	0.43	+ 0.0004 ± 0.0003	0.16
Inflammation Composite Score, ICS	*N* = 776	*N*′ = 1,313	*N* = 991	*N*′ = 1,755	*N* = 764	*N*′ = 1,292	*N* = 1,003	*N*′ = 1,776
ICS (γ_015_ for π0i)	+ 0.003 ± 0.039	0.94	+ 0.002 ± 0.037	0.96	−0.056 ± 0.046	0.22	+ 0.007 ± 0.033	0.82
ICS × Time (γ_115_ for π1i)	−0.003 ± 0.011	0.80	+ 0.007 ± 0.010	0.49	**+** **0**.**032** **±** **0**.**014**^**c**^	**0**.**019**	−0.008 ± 0.009	0.36
IL-1β	*N* = 52	*N*′ = 90	*N* = 98	*N*′ = 168	*N* = 37	*N*′ = 63	*N* = 113	*N*′ = 195
IL-1β (γ_016_ for π0i)	+ 0.007 ± 0.066	0.91	+ 0.073 ± 0.049	0.13	**+** **0**.**110** **±** **0**.**053**^**c**^	**0**.**040**	+ 0.038 ± 0.049	0.44
IL-1β × Time (γ_116_ for π1i)	+ 0.019 ± 0.028	0.51	+ 0.008 ± 0.011	0.47	+ 0.037 ± 0.025	0.13	+ 0.008 ± 0.013	0.56
IL-6	*N* = 52	*N*′ = 90	*N* = 98	*N*′ = 168	*N* = 37	*N*′ = 63	*N* = 113	*N*′ = 195
IL-6 (γ_017_ for π0i)	+ 0.008 ± 0.009^**c**^	0.36	***+*** ***0***.***018*** ***±*** ***0***.***010***	***0***.***081***	+ 0.000 ± 0.011^c^	0.99	**+** **0**.**022** **±** **0**.**008**	**0**.**006**
IL-6 × Time (γ_117_ for π1i)	−0.006 ± 0.004	0.15	+ 0.001 ± 0.003	0.65	+ 0.002 ± 0.004	0.60	−0.002 ± 0.003	0.54
IL-10	*N* = 52	*N*′ = 90	*N* = 98	*N*′ = 168	*N* = 37	*N*′ = 63	*N* = 113	*N* = 195
IL-10 (γ_018_ for π0i)	+ 0.033 ± 0.028	0.25	+ 0.065 ± 0.094	0.49	**+** **0**.**157** **±** **0**.**030**^**c,d**^	**<** **0**.**001**	***+*** ***0***.***057*** ***±*** ***0***.***033***	***0***.***077***
IL-10 × Time (γ_118_ for π1i)	−0.017 ± 0.021	0.42	−0.008 ± 0.026	0.76	**−0**.**023** **±** **0**.**009**^**c**^	**0**.**014**	+ 0.009 ± 0.021	0.65
IL-12	*N* = 52	*N*′ = 90	*N* = 98	*N*′ = 168	*N* = 37	*N*′ = 63	*N* = 113	*N*′ = 195
IL-12 (γ_019_ for π0i)	+ 0.049 ± 0.040	0.22	+ 0.049 ± 0.095	0.61	+ 0.021 ± 0.069	0.76	+ 0.064 ± 0.045	0.16
IL-12 × Time (γ_119_ for π1i)	+ 0.017 ± 0.028^**c**^	0.55	***+*** ***0***.***043*** ***±*** ***0***.***024***	***0***.***071***	+ 0.019 ± 0.019^**c**^	0.31	***+*** ***0***.***051*** ***±*** ***0***.***027***	***0***.***060***
IL-18	*N* = 52	*N*′ = 90	*N* = 98	*N*′ = 168	*N* = 37	*N*′ = 63	*N* = 113	*N*′ = 195
IL-18 (γ_020_ for π0i)	−0.000 ± 0.002	0.79	+ 0.001 ± 0.001	0.46	+ 0.004 ± 0.003	0.12	+ 0.001 ± 0.000	0.36
IL-18 × Time (γ_120_ for π1i)	+ 0.001 ± 0.001	0.36	+ 0.000 ± 0.000	0.50	−0.001 ± 0.001	0.11	+ 0.000 ± 0.000	0.15

*CES-D* Center for Epidemiologic Studies-Depression scale, *HANDLS* Healthy Aging in Neighborhoods of Diversity Across the Lifespan, *IL* Interleukin, *HS* high school, *n3* omega-3, *n6* omega-6, *PUFA* polyunsaturated fatty acids, *SEE* standard error of the estimate

^a^Models were further adjusted for other covariates (main effects and interaction with time). Time at baseline visit was set to zero. Covariates considered as potential confounders included: baseline age was centered at 50 y, sex, race, PIR, education, employment status, total energy intake centered at 2000kcal/d, total carotenoid intake at 3 mg/1,000 kcal/d, vitamin C intake at 30 mg/1,000 kcal/d, vitamin A intake at 300 RE/1,000 kcal/d, vitamin E at 3 mg/1,000 kcal/d, vitamin B-6 at 0.8 mg/1,000 kcal/d, vitamin B-12 at 3 μg/1,000 kcal/d, folate at 170 μg/1,000 kcal/d, n-3 PUFA:n-6 PUFA at 0.11. Healthy Eating Index-2010 was centered at 42, body mass index at 30, co-morbid conditions (diabetes, hypertension, dyslipidemia, CVD, inflammatory conditions) and use of NSAIDs. All these covariates were entered in models with ICS and individual component markers (i.e. hsCRP, ESR, ALBUMIN and IRON). In models with cytokines, only sociodemographic covariates (age, sex, race, PIR, education, employment status) and those that were deemed associated with the cytokines in a separate bivariate linear regression model were included. Models stratified by sex or race excluded those covariates in main and interaction effects with *TIME*

^b^*N* = number of participants in the analysis; *N*’ = total number of visits included in the analysis. Findings that were significant at a type I error of 0.05 are bolded

^c^In a separate model with interaction of inflammation marker/cytokine exposures by (sex/race) by TIME, including all other terms in the current model, *p* < 0.10 for null hypothesis that this interaction term is = 0

^d^*P* < 0.005 for exposure main effects

^e^*P* < 0.010 for interaction term (exposure × TIME)

Table 4 shows results of the association between ICS and incident EDS, across sex and race groups. Out of 1,259 subjects who were EDS-free at baseline in HANDLS, 305 incidence EDS cases were observed over a median follow-up period of 4.71 y. In the final analytic sample, EDS-free subjects were *N* = 796 of whom 185 became incident EDS cases. Parametric survival models with Weibull hazard function distribution revealed that a unit (i.e., 1 SD) increase in the ICS was associated with a 28% higher risk of incident EDS (HR = 1.28, 95% CI:1.04-1.56, *P* = 0.017) among AA only. A similar estimate was obtained from Cox PH models, while no other sex/race strata showed a significant link between ICS and incident EDS.

**Table 4:** Parametric survival model, with Weibull hazard distribution, for incident EDS *vs*. baseline ICS, HANDLS 2004–2013

	N^b^	n^b^	T^b^	Hazard Ratio	(95% CI)	*P*
*ICS*						
*All*	796	185	3,621	***1.15***	***(0.99–1.34)***	***0.068***
*Men*	348	66	1,605	1.11	(0.86–1.43)	0.44
*Women*	448	119	2,017	***1.20***	***(0.98–1.47)***	***0.073***
*Whites*	330	83	1,450	1.10	(0.86–1.41)	0.45
*African*-*Americans*	466	102	2,171	**1**.**28**	**(1**.**04–1**.**56)**	**0**.**017**

*CES-D* Center for Epidemiologic Studies-Depression scale, *HANDLS* healthy aging in neighborhoods of diversity across the lifespan, *HS* high school, *ICS* inflammation composite Score, *n3* omega-3, *n6* omega-6, *PUFA* polyunsaturated fatty acids, *SEE* standard error of the estimate

^a^Models were further adjusted for other covariates. Covariates considered as potential confounders included: baseline age was centered at 50 y, sex, race, PIR, education, employment status, total energy intake centered at 2000kcal/d, total carotenoid intake at 3 mg/1,000 kcal/d, vitamin C intake at 30 mg/1,000 kcal/d, vitamin A intake at 300 RE/1,000 kcal/d, vitamin E at 3 mg/1,000 kcal/d, vitamin B-6 at 0.8 mg/1,000 kcal/d, vitamin B-12 at 3 μg/1,000 kcal/d, folate at 170 μg/1,000 kcal/d, n-3 PUFA:n-6 PUFA at 0.11. Healthy Eating Index-2010 was centered at 42, body mass index at 30, co-morbid conditions (diabetes, hypertension, dyslipidemia, CVD, inflammatory conditions) and use of NSAIDs. Models stratified by sex or race excluded those covariates

^b^*N* = number of subjects, *n* = number of failures, T = time at risk (Person-years)

Bold values are for *P* < 0.05. Bold and italic values are for *P* < 0.10

## Discussion

This study tested the association between systemic inflammation and depressive symptoms in a prospective bi-racial cohort of urban adults. Findings suggest that serum concentrations of high-sensitivity C-reactive protein (hsCRP), z-inflammation composite score [ICS, combining elevated hsCRP and ESR with low serum albumin and iron], and serum interleukin IL- 1β were positively associated with ΔCES-Dtotal (Δ: annual rate of increase) among Whites only. IL-12 was directly related to ΔCES-Dtotal among men and AA. The race-specific associations of hsCRP, ICS, IL-1β and the sex-specific association of IL-12 with ΔCES-Dtotal were replicated for the “depressed affect” domain. Among men, lower serum albumin and higher ICS were linked with higher baseline “somatic complaints”. IL-10 among AA and IL-12 among men were inversely related to Δ“positive affect”, while “interpersonal problems” were cross-sectionally associated with IL-6 among AA and IL-10 among Whites. Finally, baseline ICS was positively associated with incident “elevated depressive symptoms” (EDS: CES-Dtotal ≥ 16) among AA (HR = 1.28, 95% CI: 1.04-1.56, *P* = 0.017).

Large epidemiologic studies published between 2004 and 2018, have found inconsistent results pertaining to associations between CRP and depression. In a longitudinal study among 5,810 participants from the Great Smoky Mountains Study, Copland et al. provided evidence for depression as a predictor of later CRP levels, but not CRP as a predictor of later depression [52]. In a cross-sectional study of 512 Morehouse and Emory Team up to Eliminate Health Disparities participants, Morris et al. found an association between CRP and the Beck Depression Inventory in White women only and this association was mediated partly by waist circumference [13]. Analyses of data from the Pittsburgh Healthy Heart Project (*N* = 263) found a weak bidirectional relationship between CRP and Beck Depression Inventory-II [11]. Furthermore, based on the ATTICA cross-sectional study involving 853 individuals, a positive correlation was observed between the Zung Self-Rating Depression Scale and CRP [3]. Using data on 1,167 male subjects from the Men Androgen Inflammation Lifestyle Environment and Stress (MAILES) Study (Australia, Asia, Europe), Tully et al. found hsCRP was associated with depression (BDI-I) and increased depressive symptoms (CES-D) in men without abdominal obesity nor metabolic risk [16]. Duivis et al. examined the longitudinal relationship between hsCRP and depressive symptoms among 667 participants from the Heart and Soul Study and found no bi-directional relationship between inflammation and depression [12]. In the longitudinal Geelong Osteoporosis Study (Australia), Pasco et al. found a direct association between hsCRP and diagnosis of MDD [53]. Other inflammatory markers besides CRP and hsCRP were also examined in relation to depression. 53 In a cross-sectional study, al-Hakeim et al. found significant increases in pro-inflamatory cytokines IL-6, IL-18, TNFα, and sIL-2R in patients diagnosed with MDD compared with the control group [15]. Finally, using data on 5978 subjects the Whitehall II study of British civil servants, Gimeno et al. found that baseline CRP and IL-6 were associated with cognitive symptoms of depression (General Health Questionnaire) at follow-up [30]. Thus, given the paucity of longitudinal studies that examined trajectories of depressive symptoms in relation to inflammatory markers and cytokines, our study adds to the body of evidence showing that in fact baseline inflammation can trigger worsening of depressive symptoms over time.

Cytokines can influence depression by dysregulating neurotransmitter synaptic availability of monoamines such as serotonin, noradrenaline and dopamine, as well as the metabolism of various amino acids such as tyrosine, tryptophan, phenylalanine and glutamate [54]. For instance, IL-1β and TNF-α can induce p38 mitogen-activated protein kinase (MAPK), which in turn can increase expression and function of serotonin reuptake pumps, leading to decreased serotonin synaptic availability leading ultimately to depressive-like behavior in laboratory animals [55]. By generating reactive oxygen or nitrogen species, proinflammatory cytokines can also decrease the availability of tetrahydrobiopterin (BH4), a key enzyme co-factor in monoamine synthesis [56]. In fact, BH4 CSF concentrations were inversely related to IL-6 CSF concentrations among patients treated with the IFN-α inflammatory cytokine [57]. Moreover, activation of indoleamine 2,3 dioxygenase (IDO) enzyme may be involved in cytokine-induced neurotransmitter alterations, partly through diverting tryptophan metabolism (a precursor of serotonin) into kynurenine, which converts to quinolinic acid, a neurotoxin produced by the activated microglia, monocytes and macrophages infiltrating the brain [58, 59]. High concentrations of quinolinic acid were found in microglia of the anterior cingulate gyrus (ACC) among suicide victims who suffered from depression [60]. Quinolinic acid directly activates the *N*-methyl-d-aspartate (NMDA) receptors while also stimulating glutamate release by blocking glutamate synaptic reuptake in astrocytes [61, 62]. Glutamate’s binding to extrasynaptic NMDA receptors can increase excitotoxicity while reducing brain-derived neurotrophic factor (BDNF) production [63]. BDNF is key to an antidepressant response partly through fostering of neurogenesis. In stress-induced animal models of depression, BDNF was shown to be markedly reduced by IL-1β and TNF-α and their downstream signaling pathways including NF-κB [64, 65]. Increased basal ganglia and dorsal ACC glutamate levels have been described in patients receiving IFN-α, while higher glutamate concentrations were associated with elevated depressive symptoms [66]. Recently, hsCRP > 3 mg/L was associated with increased basal ganglia glutamate (*vs*. a hsCRP < 1 mg/L) that correlated with anhedonia and poor psychomotor speed among depressed patients [67].

The study has several notable strengths. First, its longitudinal design improves the ability to ascertain temporal relationships between the exposure of systemic inflammation and the outcome of depressive symptoms. Second, the large final analytic sample size allowed for further stratification by key sociodemographic factors, namely sex and race without significant decline in statistical power. Third, the intercept and slopes in the mixed-effects regression models were adjusted for numerous potentially confounding covariates. Nevertheless, several limitations should also be noted, such as potential non-participation selection bias, the effect of which was minimized by the use of a 2-stage Heckman selection model. Furthermore, while inflammatory markers were direct blood level measures, our outcome of interest was self-report which could be a source of measurement error. Given the observational nature of the study, residual confounding cannot be ruled out. Finally, although no bi-directional analysis was done, this can potentially be carried out in a future study.

## Conclusion

In sum, several markers of systemic inflammation and cytokines were shown to be linked to depressive symptoms’ trajectory over time, differentially across sex and race groups, with each exposure being predictive of baseline and rates of change of specific depressive symptom domains. While a composite score of inflammation (ICS) was found to be linked with a faster trajectory of CES-D scores and domains for the most part among Whites, ICS was also directly related to incident EDS among AA. Given those inconsistent race-specific findings between annual rates of change *vs*. incident binary outcomes, additional longitudinal studies in similar large and diverse samples are needed.

## Supplementary information

OSM 1 and 2
