# Supplementary material for: Systemic inflammation is associated with depressive symptoms differentially by sex and race: a longitudinal study of urban adults
Source: Mol Psychiatry. 2019 Apr 24;25(6):1286–300. doi: 10.1038/s41380-019-0408-2 (PMC6813878; doi:10.1038/s41380-019-0408-2)
Supplement: Supplementary file 1 — OSM 1 and 2 [file 41380_2019_408_MOESM1_ESM.docx]

OSM 1: Covariate vs. exposure associations: bivariate linear regression

| **CRP** | **ESR** | **Albumin** | **Iron** | **Inflammation composite** | **Covariates** |
| --- | --- | --- | --- | --- | --- |
| + | + | - | + | + | Age |
| - | - | + | + | - | Sex |
| + | + | - | - | + | Race |
| - | - | + | + | - | Pir (0=below, 1=above poverty) |
| edubr=2, 0; edubr=3, -; edubr=9, 0 | 0 | 0 | 0 | 0 | Education |
| 0 | smoke=1, -; smoke=9, 0 | smoke=1, -; smoke=9, 0 | 0 | 0 | Smoking |
| currdrugs=1, -; currdrugs=9, 0 | currdrugs=1, -; currdrugs=9, 0 | currdrugs=1, 0; currdrugs=9, - | currdrugs=1, +; currdrugs=9, 0 | currdrugs=1, -; currdrugs=9, 0 | Drug use |
| - | - | + | employed=1, +; employed=9, 0 | - | Employment status (0=unemployed, 1=employed) |
| - | - | 0 | + | - | Energy intake |
| 0 | 0 | 0 | 0 | 0 | Carotenoids |
| 0 | 0 | 0 | 0 | 0 | Vitamin C |
| 0 | + | 0 | 0 | 0 | Vitamin A |
| 0 | 0 | + | 0 | - | Vitamin E |
| 0 | 0 | + | 0 | - | Vitamin B-6 |
| + | + | - | - | + | BMI |
| 0 | 0 | + | 0 | - | Folate |
| 0 | 0 | 0 | 0 | 0 | B-12 |
| 0 | 0 | + | 0 | - | Healthy Eating Index |
| 0 | 0 | 0 | 0 | 0 | N3/n6 PUFA ratio |
| + | + | - | - | + | Diabetes |
| + | + | - | - | + | Hypertension |
| 0 | + | 0 | 0 | 0 | Dyslipidemia |
| + | + | - | - | + | CVD |
| 0 | + | - | - | + | Inflammatory conditions |
| 0 | + | - | - | + | NSAIDS |

-: negative significant association, +: positive significant association, 0=null association

OSM 1 Continued: Covariate vs. exposure associations: bivariate linear regression

| **IL-1b** | **IL-6** | **IL-10** | **IL-12** | **IL-18** | **Covariates** |
| --- | --- | --- | --- | --- | --- |
| 0 | 0 | 0 | 0 | 0 | Age |
| 0 | 0 | 0 | 0 | 0 | Sex |
| 0 | + | 0 | 0 | 0 | Race |
| 0 | 0 | 0 | 0 | - | Pir (0=below, 1=above poverty) |
| 0 | 0 | 0 | 0 | 0 | Education |
| smoke=1, 0; smoke=9, + | smoke=1, +; smoke=9, 0 | 0 | 0 | smoke=1, +; smoke=9, 0 | Smoking |
| 0 | 0 | currdrugs=1, 0;  currdrugs=9, + | currdrugs=1, 0;  currdrugs=9, + | 0 | Drug use |
| 0 | employed=1, -; employed=9, 0 | 0 | 0 | employed=1, -; employed=9, 0 | Employment status (0=unemployed, 1=employed) |
| 0 | 0 | 0 | 0 | 0 | Energy intake |
| 0 | 0 | 0 | 0 | 0 | Carotenoids |
| 0 | 0 | 0 | 0 | 0 | Vitamin C |
| 0 | 0 | 0 | 0 | 0 | Vitamin A |
| 0 | 0 | 0 | 0 | 0 | Vitamin E |
| 0 | 0 | 0 | 0 | 0 | Vitamin B-6 |
| 0 | 0 | 0 | 0 | 0 | BMI |
| 0 | 0 | 0 | 0 | 0 | Folate |
| 0 | 0 | 0 | 0 | 0 | B-12 |
| 0 | 0 | 0 | 0 | 0 | Healthy Eating Index |
| 0 | 0 | 0 | 0 | 0 | N3/n6 PUFA ratio |
| 0 | 0 | 0 | 0 | 0 | Diabetes |
| 0 | 0 | 0 | + | 0 | Hypertension |
| 0 | 0 | 0 | 0 | 0 | Dyslipidemia |
| 0 | + | 0 | 0 | 0 | CVD |
| 0 | 0 | 0 | 0 | + | Inflammatory conditions |
| 0 | 0 | 0 | 0 | 0 | NSAIDS |

-: negative significant association, +: positive significant association, 0=null association

**OSM 2: Mixed-effects regression models**

The mixed-effects regression models can be summarized as follows:

**Multi-level models** vs. **Composite models**

| **Eq.**  **1.1-1.4** | $Y_{ij}=\pi_{0i}+\pi_{1i}Time_{ij}+\varepsilon_{ij}$ |  |  |
| --- | --- | --- | --- |

Where Y_ij_ is the outcome (CES-D total or domain-specific score) for each individual “i” at visit “j”; $\pi_{0i}$is the level-1 intercept for individual i; $\pi_{1i}$is the level-1 slope for individual i; $\gamma_{00}$is the level-2 intercept of the random intercept $\pi_{0i}$; $\gamma_{10}$is the level-2 intercept of the slope $\pi_{1i}$; $Z_{ik}$is a vector of fixed covariates for each individual *i* that are used to predict level-1 intercepts and slopes and included baseline age (Age_base_) among other covariates. X_ija_, represents the main predictor variables (CRP, ESR, ALB, IRON, ICS, IL-1β, IL-6, IL-10, IL-12, IL-18); $\zeta_{0i}$and $\zeta_{1i}$are level-2 disturbances; $\varepsilon_{ij}$is the within-person level-1 disturbance. Of primary interest are the main effects of each exposure X_a_ (γ_0a_) and their interaction with *TIME* (γ_1a_), as described in a previous methodological paper.^1^

**References**

1. Blackwell E, de Leon CF, Miller GE. Applying mixed regression models to the analysis of repeated-measures data in psychosomatic medicine. *Psychosom Med* 2006; **68**(6)**:** 870-878.
